# diffGEK: differential gene expression kinetics

**DOI:** 10.1093/bioinformatics/btaf316

**Published:** 2025-06-10

**Authors:** Melania Barile, Shirom Chabra, Tomoya Isobe, Berthold Gottgens

**Affiliations:** Department of Haematology, Wellcome–MRC Cambridge Stem Cell Institute, University of Cambridge, Jeffrey Cheah Biomedical Centre, Cambridge CB2 0AW, United Kingdom; Centre for Translational Stem Cell Biology, HKSTP, Hong Kong SAR, China; Department of Haematology, Wellcome–MRC Cambridge Stem Cell Institute, University of Cambridge, Jeffrey Cheah Biomedical Centre, Cambridge CB2 0AW, United Kingdom; Department of Haematology, Wellcome–MRC Cambridge Stem Cell Institute, University of Cambridge, Jeffrey Cheah Biomedical Centre, Cambridge CB2 0AW, United Kingdom; Department of Haematology, Wellcome–MRC Cambridge Stem Cell Institute, University of Cambridge, Jeffrey Cheah Biomedical Centre, Cambridge CB2 0AW, United Kingdom

## Abstract

**Motivation:**

A defining characteristic of all metazoan organisms is the existence of different cell states or cell types, driven by changes in gene expression kinetics, principally transcription, splicing and degradation rates. The RNA velocity framework utilizes both spliced and unspliced reads in single cell mRNA preparations to predict future cellular states and estimate transcriptional kinetics. However, current models assume either constant kinetic rates, rates equal for all genes, or rates completely independent of progression through differentiation. Consequently, current models for rate estimation are either underparametrized or overparametrized.

**Results:**

Here, we developed a new method (diffGEK) which overcomes this issue, and allows comparison of transcriptional rates across different biological conditions. diffGEK assumes that rates can vary over a trajectory, but are smooth functions of the differentiation process. Analysing Jak2 V617F mutant versus wild type mice for erythropoiesis, and Ezh2 KO versus wild type mice in myelopoiesis, revealed which genes show altered transcription, splicing or degradation rates between different conditions. Moreover, we observed that, for some genes, compensatory changes between different rates can result in comparable overall mRNA levels, thereby masking highly dynamic changes in gene expression kinetics in conventional expression analysis. Collectively, we report a robust pipeline for comparative expression analysis based on altered transcriptional kinetics to discover mechanistic differences missed by conventional approaches, with broad applicability across any biomedical research question where single cell expression data are available for both wild type and treatment/mutant conditions.

**Availability and implementation:**

This study does not include new data. All the codes are available on github: https://github.com/mebarile/transcriptional_kinetics.

## 1 Introduction

There is no doubt that single-cell trancriptomic profiling represents a transformative technique to interrogate differentiation processes. By providing a snapshot of the RNA expression on an individual cell level, across a population of thousands of cells, each at distinct points in the dynamic process under study, differentiation trajectories can be reconstructed. These trajectories are inherently dynamic, characterized by changes in both the programmes and magnitudes of gene expression, as cells progress through a repertoire of cell states. Importantly, varying conditions—whether cell intrinsic, such as genetic mutations, or cell extrinsic, such as inflammation or pharmacological manipulation—can induce altered differentiation processes. Understanding the biology of how distinct conditions drive differing trajectories is crucial for understanding disease pathogenesis, drug mechanisms and stem cell engineering protocols.

The high resolution of scRNA-seq, combined with the wealth of transcriptomic information it provides, presents an opportunity to disentangle heterogeneity in differentiation processes between conditions, and unravel the mechanistic underpinnings that drive these differences. Differential gene expression (DGE) remains the predominant method to bridge the gap between observable phenotypes and the corresponding molecular mechanisms, evidenced by DGE often being the final (or near final) step in scRNA-seq analysis pipelines. A key problem, however, is that the majority of statistical or computational analysis procedures to achieve this either (i) fail to exploit the continuous resolution provided by scRNA-seq trajectories, or (ii) focus exclusively on the abundance of spliced mRNA, which limits interpretability of the underlying biological changes.

In fact, despite the continuous nature of differentiation trajectories, DGE techniques predominantly adopt a discrete approach. More specifically, gene expression is often compared on discrete groups of cells in the differentiation pathway, e.g. by comparing clusters of cells by means of *T*-test based methods or upon modelling gene expression variance as a function of expression average (Love *et al.* 2014, Robinson *et al.* 2010). Critically these discrete approaches fail to account for the continuous dynamics of gene expression changes. Moreover, the comparison of discrete cell clusters within or between lineages introduces interpretative challenges. In particular, ambiguity arises in determining: which clusters should be compared, the proper aggregation of results from various pairwise cluster comparisons at different trajectory points, or how to account for the non-independence of these comparisons. A few methods, such as tradeSeq (Van den Berge *et al.* 2020), GPfates (Lönnberg *et al.* 2017), and Monocle 2 (Qiu *et al.* 2017), have been developed to improve trajectory-based differential expression analysis by adopting a dynamic approach. These methods process the continuous gene expression profiles obtained by ordering cells according to their transcriptomics difference from a pre-defined root cell (pseudotime), and compare these profiles across conditions, on a gene-by-gene level to determine differential expression. While these techniques better account for the dynamic nature of differentiation, a caveat is that they only rely on the abundance of spliced mRNA transcripts. More recent work by Tiberi *et al.* (2024), on the other hand, has leveraged the unspliced transcript abundance to uncover changes among experimental conditions. This method, however, does not provide interpretability into the changes in underlying transcription, splicing and degradation rates (TSDr) which govern the process of gene expression. Moreover, since gene expression is an emerging property of these rates, discrepancies among conditions may exist at the rate level without precipitating as variations in spliced mRNA abundance; such differences would be ‘missed’ when comparing conditions using DGE. Hence, methods to infer TSDr profiles within trajectories and compare these between conditions are vital to further advance the field.

To derive TSDr from scRNA-seq data from common scRNA-seq protocols, without the need for supplementary experiments or alterations to sequencing methodologies, we leveraged the combination of spliced and unspliced transcript abundance and build upon the foundations of the RNA velocity framework. Up until now, the relative abundance of nascent (unspliced) and mature (spliced) transcripts have been utilized as a powerful feature to interrogate the future state of a cell and the pseudotemporal order of differentiation in a lineage. These analyses are rooted in the widely-recognized RNA velocity framework (La Manno *et al.* 2018, Bergen *et al.* 2020). The core premise of RNA velocity is that spliced and unspliced count dynamics are coupled and governed by three kinetic parameters: transcription, splicing, and degradation rates. While effective in simple systems, the original framework falls short in certain scenarios. For example, if the nascent transcript is noisy, analysis is affected and several autoencoder-based methods have been suggested to infer a common latent space that reduces noise and allows correct trajectory inference (Qiao and Huang, 2021). Another work has suggested to use a per gene latent time (Gao *et al.* 2022) to account for gene specific dynamics. Still, these methods assume that the kinetic rates remain fixed across the differentiation trajectory (with the possibility to switch on/off the transcription), and thus do not account for conspicuous kinetics change along a trajectory, typical of development and particularly erythropoiesis (Barile *et al.* 2021). To address this limitation, we advocate for the incorporation of cell-specific values for these rates, similarly to what has been proposed earlier (Qiu *et al.* 2022, Li *et al.* 2024), but with further constrains to reduce the huge dimensionality of the respective unknown parameter space.

This article introduces diffGEK, a method for analysing trajectory-based DGE kinetics in scRNA-seq data. By extending the RNA velocity framework, diffGEK initially estimates per-cell and per-gene kinetic parameters using known lineage and pseudo-temporal ordering of cells for a specific condition. Additionally, diffGEK integrates a statistical strategy to discern whether a gene exhibits differential kinetics between any two biological conditions, across all possible permutations (Table 1). Evaluation of the pipeline on simulated data and its application to two murine haematopoietic stem cell differentiation datasets demonstrates diffGEK’s utility as a complementary tool to DGE analysis. diffGEK introduces an additional perspective for unravelling heterogeneity between states, enhancing interpretability and fostering a more nuanced understanding of the underlying biology.

**Table 1.** Schematic of the eight possible models used to fit the data.^a^

	α	β	γ	k
M1	Same	Same	Same	32
M2	Any	Same	Same	40
M3	Same	Any	Same	40
M4	Same	Same	Any	40
M5	Any	Any	Same	48
M6	Any	Same	Any	48
M7	Same	Any	Any	48
M8	Any	Any	Any	56

a ‘Same’ means that the corresponding rate is forced to be the same across conditions. ‘Any’ means that it can be different. α: transcription rate; β: splicing rate; γ: degradation rate; k: number of model parameters (eight spline nodes per rate, four initial conditions and four estimated errors).

## 2 Materials and methods

### 2.1 General approach

To model the spliced and unspliced counts over pseudotime we relied on the previously established coupled ODE system:


(1) $$\begin{array}{c} {\dot{U}}_{g}(t)=\alpha_{g}(t)-\beta_{g}(t)\;U_{g}(t)\\ {\dot{S}}_{g}(t)=\beta_{g}(t)\;U_{g}(t)-\gamma_{g}(t)\;S_{g}(t) \end{array}$$

where $U_{g}(t)$ and $S_{g}(t)$ are the number of unspliced and spliced counts for gene *g* at pseudotime *t*, respectively; $\alpha_{g}(t)$, $\beta_{g}(t)$ and $\gamma_{g}(t)$ are the kinetic parameters (transcription, splicing and degradation rate, respectively) for gene *g* at pseudotime *t*. Compared to other articles, we modelled the kinetic parameters in a novel way. First, the parameters are gene specific. Second, they are a function of the cellular state, that is, of the pseudotime. Since this would imply a big number of unknown variables to estimate, we further assumed that the parameters can be modelled with natural cubic splines, and required that such splines are as least wobbly as possible (see Section 2.3).

### 2.2 Gene selection

In our article, we used the top HVGs to run the analysis, as chosen after filtering genes that do not have enough spliced and unspliced counts, according to the scVelo filtering step in the pipeline. Notice that this step is not optional. Noisy genes affect the analysis and the statistical power. We recommend to use no more than 2000 top genes.

### 2.3 Optimization

To model $\alpha_{g}(t)$, $\beta_{g}(t)$ and $\gamma_{g}(t)$, we chose natural cubic splines with eight nodes per each parameter. Furthermore we included two more variables to account for the initial conditions, and two more to account for the estimated error on each time course (spliced and unspliced counts over pseudotime). So in total we have 28 unknown variables per gene. We calibrated the model output to the simulated or real data pooled in 20 values of the pesudotime. So for each dataset, each pool contains a number of cells corresponding to the dataset size divided by 20 (floored value). The values used for fit are the averaged values of the spliced/unspliced counts, and the averaged values of the corresponding pseudotimes. For simulations, we generated the counts directly. For real data, we imputed the normalized counts with the standard scVelo pipeline. For each gene independently, we maximized the following negative loglikelihood function:


(2) $$\begin{array}{c} L=-\frac{1}{2}\sum_{i=1}^{20}(\;\text{log}(2\;\pi \;\sigma_{s})+\frac{{\left(S_{o,g,i}-S_{g,i}\right)}^{2}}{\sigma_{g,s}}+\\ \;\text{log}(2\;\pi \;\sigma_{g,u})+\frac{{\left(U_{o,g,i}-U_{g,i}\right)}^{2}}{\sigma_{g,u}})-\\ \rho \;\sum_{i=1}^{7}({\left(\alpha_{g,i+1}-\alpha_{g,i}\right)}^{2}+\\ {\left(\beta_{g,i+1}-\beta_{g,i}\right)}^{2}+{\left(\gamma_{g,i+1}-\gamma_{g,i}\right)}^{2}) \end{array}$$

where $U_{o,g,i}$ and $S_{o,g,i}$ are the measured pooled values of the unspliced and spliced counts respectively for gene *g* at the pooled pseudotime value *i*; $U_{g,i}$ and $S_{g,i}$ are the predicted values of the unspliced and spliced counts respectively for gene *g* at the pooled pseudotime value *i*; $\sigma_{g,s}$ and $\sigma_{g,u}$ are the estimated variance on the all time course of the spliced and spliced counts for gene *g* respectively; ρ is a parameter (to be fixed with simulations) regularizing the wobbliness of the splines; for the kinetic rates, the sum runs over the splines nodes. In case of matched replicates, diffGEK can also be run in ‘samples’ mode. In this case, the likelihood function above will have *nrep* as many terms, where *nrep* is the number of replicates; each term in the likelihood will correspond to the data in one sample as taken separately. The function needs to be rewritten depending on the number of samples in a specific experimental design. Examples are found on the github page. Of note, the ‘samples’ mode, in the way we designed it, still neglects the sample-to-sample variability. Incorporating the latter, however, is still possible upon modifying the model building codes. Overall, the likelihood function has three terms. One term accounts for the fit accuracy, and it assumes normal distribution because we pooled all the datapoints for a certain condition in just 20 points, upon averaging the counts and the corresponding pseudotime values. This means that the average was performed among many cells per each point, which justifies the normality assumption. Another term accounts for the estimated error. The error was estimated because it helps comparing data of different magnitudes, which can be the case sometimes for gene counts even if we lognormalized the data (within the same trajectory, a gene can go from 0 to thousands of counts). Then, the corresponding likelihood term derives again from the normality assumption (upon deriving the density of the Gaussian distribution with respect to the estimated errors). Finally, the third term is a regularization parameter that forces the splines to be as straight as possible (Fischer *et al.* 2019). The optimization was performed in MATLAB 2019 with the toolboxes AMICI and PESTO. For each gene, we launched 3000 optimizations starting from randomly picked initial points. Constrains on parameters were as follows:

- Kinetic parameters: from 0 to 6.7 per unit of pseudotime;
- Estimated initial values: from 10% to 300% of the measured initial value;
- Estimated errors: from 1% to 10% of the maximum value of the corresponding data.

Note that all the parameters must be positive and do not in principle need an upper/lower bound for the estimation. The reason for which we chose to add bounds is for optimization speed. The values were selected empirically, starting from a small interval and then widening the interval until the model converged to a value sitting comfortably in the interval.

### 2.4 Model selection

The framework described so far allows the estimation of the kinetic parameters in units of the pseudotime. While this can be informative on the variation of the rates along a trajectory, it is also difficult to interpret given that the pseudotime unit is arbitrary. We can nevertheless gain information of what rates change among different conditions. For a comparison among two conditions, for example, there can be eight possible scenarios (see Table 1). We fit these eight models simultaneously to the spliced and unspliced counts for each condition; for each gene, each of the eight models is optimized independently. After optimization, for each genes we obtain the estimated parameters for each of the eight models. In order to choose the best model, we first need to evaluate the goodness of the fit. We found empirically that the value of the negative loglikelihood is not good to compare different models, because the error per time course is estimated independently and can be very different across models (neither it helps to force such error to be the same, given that the values for the two conditions can be very different). We thus used a squared sum of residuals, $SSR_{g,m}$, weighted by an extra parameter, μ, that has again to be determined via the simulations (see Section 2.5). We then ranked models according to their corrected Akaike index per gene *g* per model *m*, $A_{g,m}$, which is calculated as follows: $A_{g,m}=2k_{m}+SSR_{g,m}+(2k_{m}^{2}+2k_{m})/(n-k_{m}-1)$, where $k_{m}$ is the number of parameters and *n* the fixed number of data (40 for all the example in this article). The model with the minimum $A_{g,m}$ per each gene is picked as the best model.

### 2.5 Simulations

In order to determine the values of ρ and μ, we simulated 100 genes for each of the 8 models described in the previous section. The genes are simulated with randomly extracted values of the initial conditions and of the first node of the parameters’ splines. The following nodes are extracted in a Gaussian range of the previous node, to avoid massive wobbling in the parameters’ values. The corresponding 800 sets of 4 time courses (spliced and unspliced time courses for each of the two conditions) are re-fit, each with the 8 possible models, for a total of 64 000 fits. For each gene simulated with model *m*, we would of course expect to recover model *m* as best model, while recovering a different model can imply having a certain number of true/false positive/negative outcomes. By false positive we mean any rate that is simulated as non-changing but for which the selected model predicts a change among conditions, and so on (Box 2). We found that the values for ρ=10 and μ=0.01 maximized the true positive and true negative while keeping the false positive and negative low. We used this values for running our model on real data.

### 2.6 Jak2

The Jak2 landscape was taken from Isobe *et al.* (2023). We selected the erythroid trajectory because we expect Jak2 to produce changes in this lineage. We ran diffGEK on the top 1000 HVGs as selected by the scVelo command scv.pp.filter_and_normalize, plus the tradeSeq differentially expressed genes as found by the authors in the original article, for a total of 1048 genes. Note that the diffGEK pipeline just needs the HVGs, but we also included DE genes as a way to double-check the tool performance.

### 2.7 Ezh2

The Ezh2 dataset was taken from Liu *et al.* (2021), accession GSE179084. We downloaded 6 raw fastq data (GSM4711730, GSM4711731, GSM4711732, GSM4711739, GSM4711740, GSM4711741) corresponding to the 3 time points for WT and Ezh2-KO conditions; we ran cellranger and then velocyto to obtain spliced/unspliced counts. We then created an UMAP following the standard scanpy pipeline, and identified myeloid progenitors with CellRank (Lange *et al.* 2022). We then ran tradeSeq to get differential trajectories for the genes at all time points together and for each time point separately. We ran diffGEK on all such genes plus 1000 top HVGs, for a total of 1228 genes. Note that the diffGEK pipeline just needs the HVGs, but we also included DE genes as a way to double-check the tool performance.

### 2.8 Ontology analysis

For both Jak2 and Ezh2 we performed ontology analysis on the EnrichR website. For both cases, we used the genes that have differential kinetics, and took the BioPlanet gene set. The adjusted maximum *P-*values are specified in the respective figure legends.

### 2.9 Running the pipeline

diffGEK is a collection of MATLAB scripts. The order of the scripts and the explanation on how to run them can be found on our Github: https://github.com/mebarile/transcriptional_kinetics. There is a vignette explaining step by step what each code does, and all the codes that we used for this manuscript. In brief, diffGEK specific codes do not need to be modified unless the experimental design is very different from what we used, such as there are three instead of two conditions, or very short/long trajectories. Users can also modify the scripts if they want to increase resolution of the splines or include sample to sample variability. Regularization parameters do not need to be re-optimized unless, again, the users want to, or think that their experimental design is too different from the one we used in our article.

### 2.10 Runtime information

diffGEK is computationally intense, not because of high memory requirement, but because of the large number of models that has to be fit to the genes. Time depends on the dataset, but this is an idea to predict what to expect: A normal gene can require a few minutes for 300 different initial conditions. We ran 100 genes on a CPU node (56 cores, each with 3.5 GB memory) sequentially. We ran on a remote server to send many runs in parallel, taking around 5  for each model. Also, the models can be parallelized, but we chose to run: model1, then models 2–4, then models 5–7, then model 8; for a total of two days of computation for a dataset with around 1000 genes.

## 3 Results

### 3.1 Simulations

**Figure 1.**
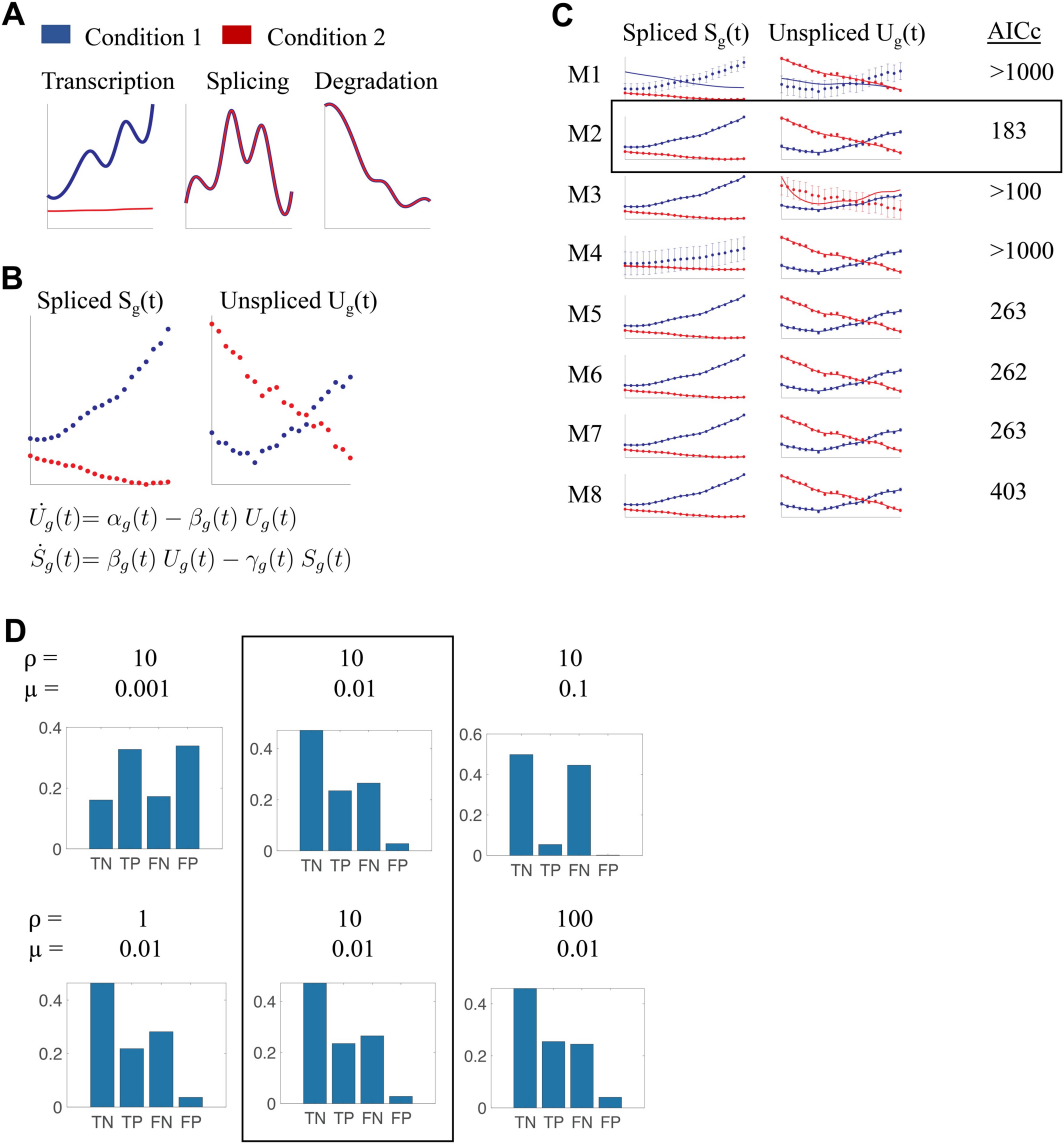
Schematic of diffGEK and the simulations performed to optimise it (A) Generating the kinetic rates. The panel show an example of rates generated for two conditions (in blue and red) for a gene regulated according to Model 2: different Transcription rate; for the Splicing and Degradation rates, the assumption is that the rates are the same, so the curves overlap. (B) Generating spliced and unspliced counts. Once the kinetic parameters have been generated, the unspliced and spliced counts are computed with the usual RNA velocity equations. (C) Each gene is refitted with all eight possible models. Dots: generated data, equal for all panels to allow model selection on the same data. Error bars: errors as estimated by each model in the fitting procedure.Continuous lines: model fit. Not all models fit well the data, hence the model selection: the model corresponding to the smallest corrected Akaike Information Criteria index (AICc) is selected as best model for the corresponding gene and regularisation parameters (black rectangle). (D) For each pair of regularization parameters, we compute the number of rates that are correctly/incorrectly predicted. TN: True Negative; TP: True positive, FN: False Negative, FP: False Positive. The pair that gives the best compromise between TN, TP, FP, FN is selected (black rectangle). The diffGEK pipeline is ready.

We performed systematic simulations to prove that our framework can classify genes whose kinetics are different among two conditions and to optimize the fitting and classification procedure (Fig. 1). Since any of the three kinetic rates can be identical or different among the two conditions, we have in total $2^{3}=8$ possible configurations, which we refer to as models. For each of these eight models (M1-8, Table 1), we simulated one hundred gene dynamics according to the following steps. First, we generated per-cells values of the three kinetic rates (transcription, splicing and degradation), modelling them with eight-nodes natural cubic splines and in such a way to keep their values similar among nodes to avoid oscillating patterns (Fig. 1A, Section 2). Modelling the rates as functions of pseudotime accounts for the potential changes in kinetics along a trajectory. Then, we generated the values of the spliced and unspliced counts (Fig. 1B) for each model and condition with the standard ODE system describing RNA velocity (La Manno *et al.* 2018, Bergen *et al.* 2020) at twenty predefined pseudotime points, but using the pre-generated cell-dependent rates. The corresponding eight hundred genes were refitted each with the eight possible models, and with three different values of a penalization parameter (ρ, Fig. 1C and 1D), which ensures that the splines are not wobbly, for a total of 19 200 fits (see also Fig. 1, available as supplementary data at *Bioinformatics* online for a more detailed example of the effects of the two regularization parameters). Successively, we ranked the models per each gene according to their corrected Akaike Information Criterion (AICc, Fig. 1C), which accounts for the number of unknown variables and the square sum of residuals, as weighted by an extra regularization term, μ, which was allowed to take three different values (Fig. 1D and Section 2). Depending on the values of the regularization terms, our procedure succeeds or fails to classify the ground truth (see e.g. Table 2). In general, we saw that it is very easy to classify M1 genes as those genes that were generated with Model 1, that is, assuming no differential kinetics. It was also generally easy to correctly classify those genes for which only one kinetic parameter changes. It was more complicated, though, to ensure that genes with more than two parameters changing among conditions were not classified as genes with only one parameter changing. We thus scored the performance of our procedure based on how many rates were correctly classified with respect to the ground truth (Table 3 and Section 2). In total there are four possible scenarios for the rates: True Positive (TP), False Negative (FN), False Positive (FP) and True Negative (TN). Figure 1D shows the histograms of TP, TN, FP and FN classifications for each combination of regularization parameters. We picked the combination ρ=10 and μ=0.01 to ensure a large number of TP and TN, and a small number of FP, even if this means trading a large number of FN. Once picked the regularization parameters, we have automatically the best prediction for the model used to generate each gene. Overall, we created a pipeline that can infer genes that have differential kinetics among two conditions and dubbed it diffGEK.

**Table 2:** Example of classification of the 100 genes generated with each of the models on the rows, then refitted with the models in the columns, for a fixed set of regulatory terms. Perfect classification or non-significative classification is achieved in most cases where at most one parameter change among conditions, but it is more difficult when more than one parameter change. It is so needed to tune the regulatory terms to achieve the best deal among false/true positive/negative.

	M1	M2	M3	M4	M5	M6	M7	M8
M1	100	0	0	0	0	0	0	0
M2	47	46	4	3	0	0	0	0
M3	42	2	52	4	0	0	0	0
M4	19	3	2	76	0	0	0	0
M5	21	24	43	5	2	1	4	0
M6	10	18	7	49	8	4	4	0
M7	8	4	22	41	6	11	8	0
M8	3	14	15	34	7	15	12	0

**Table 3:** Numbers of True Negative (TN), True Positive (TP), False Negative (FN) and False Positive (FP) rates. A gene generated with Models M1-8 (rows) is fitted with models M1-8 (columns). Depending on which model is picked as best model, there can be either 0, 1, 2 or 3 TN, TP, FP, FN rates (clockwise starting from top left in the group of 4 numbers at the intersection of each pair model used for generating vs model used for fitting).

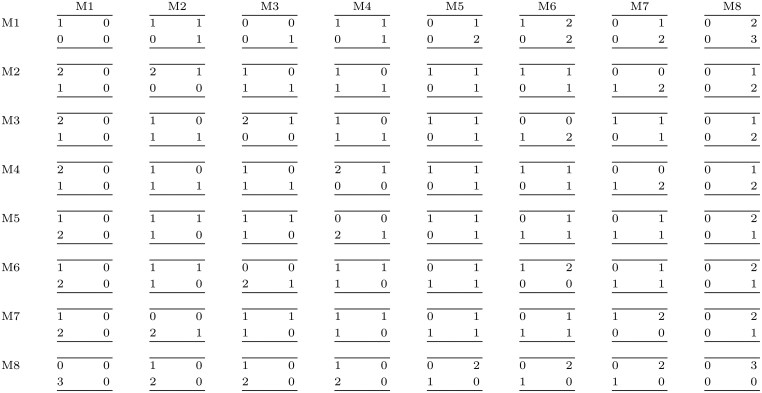

### 3.2 Transcriptional kinetics in Jak2 V617F mutant mice erythropoiesis

**Figure 2.**
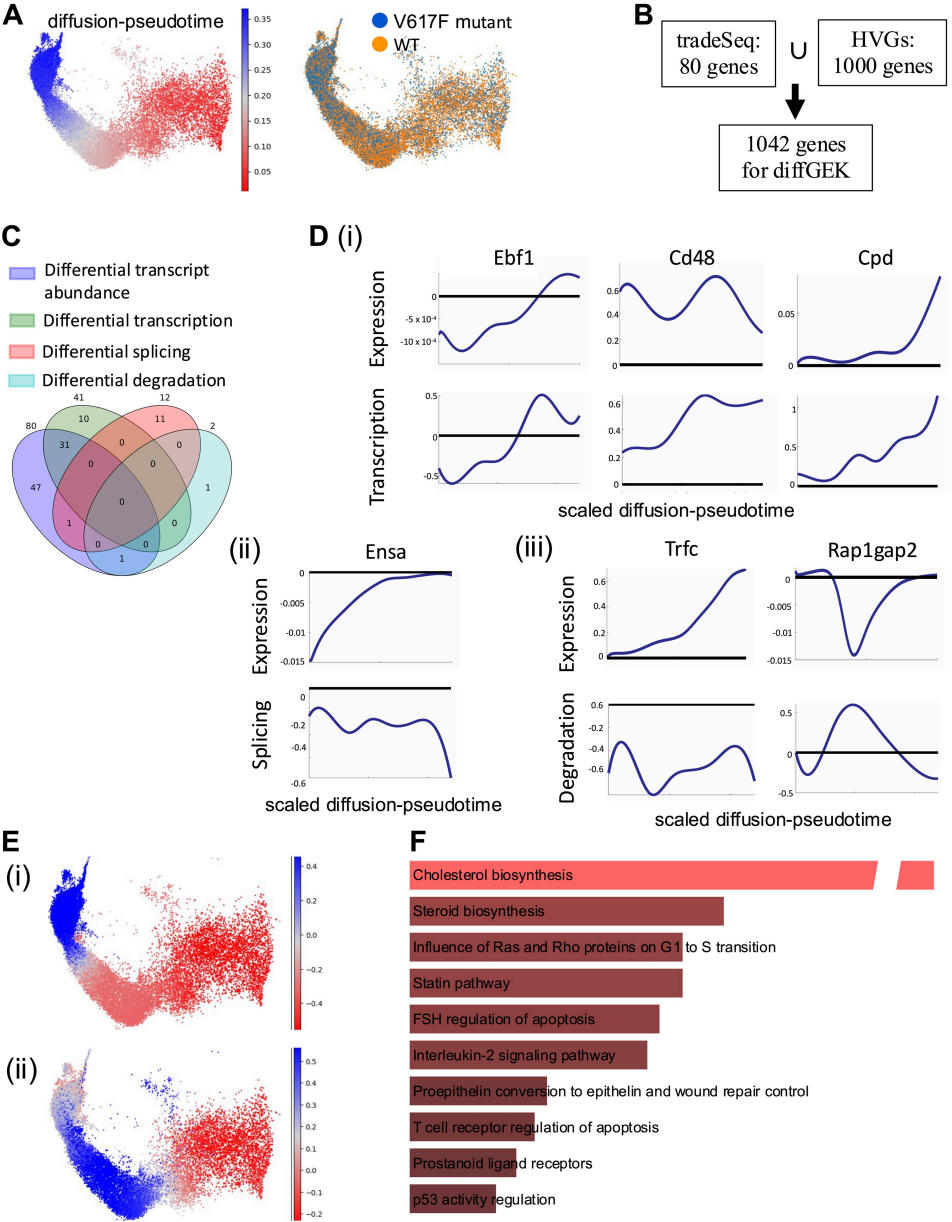
Application of the diffGEK pipeline to erythropoiesis in Jak2 V617F mutant vs WT mice (A) UMAP showing diffusion pseudotime and conditions as adapted from the original manuscript (Isobe *et al*. 2023). (B) Choice of genes for the diffGEK pipeline. The diffGEK pipeline needs the HVGs, but we also included DE genes as a way to double-check the tool performance. (C) Overlap between the differentially expressed genes and the diffGEK significant genes, sorted by rate type. (D) (i-iii) Examples of genes with differential kinetic rates (transcription, splicing or degradation respectively). For each gene, the panels represent the log2 fold change between the mutant and the wild type expression (top) and the rates (bottom). (E) Example of the log2 fold change of the transcription rate of Ebf1 (i) and the degradation rate of Rap1gap2 (ii). (F) Gene ontology of the diffGEK significant genes (that is, all but those classified as M1). Source: EnrichR, BioPlanet gene set. Adjusted *P-*values < 0.01.

To evaluate diffGEK on biological data, we first applied the pipeline to a scRNA-seq dataset encompassing adult murine haematopoiesis from paired wild-type and homozygous Jak2 V617F mutant mice (Isobe *et al.* 2023). Homozygous Jak2 V617F mutations induces alterations in erythroid differentiation, resulting in a higher accumulation of mature erythroid cells in comparison to the paired wild-type controls (Fig. 2A). Based on this understanding, we narrowed down our analysis to cells belonging to the erythropoietic trajectory, as determined by the cell fate probabilities computed by the original authors. Overall, the erythroid trajectory input into diffGEK consisted of 24 154 cells, derived from three sets of paired mutant and wild type mice. Of note, this dataset was deeply sequenced, with a median of 14 389 transcripts per cell, enabling reliable capture of unspliced reads. To input into the diffGEK pipeline, we integrated the top 1000 highly variable genes (HVGs) alongside the 80 genes identified as differentially expressed along the trajectory via tradeSeq, resulting in a collective set of 1042 genes due to list overlap (Fig. 2B and Section 2). Among these genes, 55 exhibited differential kinetics: 41 differed in transcription, 12 in splicing, and 2 in degradation rates (Fig. 2C). In this Jak2 V617F mutation context, genes demonstrating DGE kinetics displayed alterations in a single parameter only, rather than combinatorial parameter changes. Hypergeometric test shows that the overlap of genes that have differential kinetics and are differentially expressed according to tradeSeq is significative (p≪0.001). For such genes exhibiting differential expression alongside changes in gene expression kinetics, diffGEK offers explanatory insights into the kinetic parameters driving the altered gene expression profile in the mutant (Fig. 2D). Furthermore, since diffGEK adopts a continuous approach that is rooted in pseudotime, the output can be used to pinpoint the stages of differentiation, and the corresponding cell types, where kinetic rate differences are most pronounced (Fig. 2E).

To gain further biological insight, we explored the gene ontology of genes displaying differential TSDr. Pathway enrichment analysis of these genes revealed a substantial overlap with pathways independently enriched from differentially expressed genes (Fig. 2F). This highlights that diffGEK captures relevant differences between biological states, aligning with observations made by widely-utilized techniques. Key pathways exhibiting high enrichment scores included: (i) Cholesterol Biosynthesis and Steroid Biosynthesis, (ii) the p53 pathway, and (iii) the G1 to S transition. Notably, these pathways have been reported to play regulatory roles in erythroid differentiation, and were also enriched amongst differentially expressed genes. Cholesterol and steroid metabolism have recently been reported to control the balance between erythroid proliferation and differentiation, by regulating the cell cycle and induction of globin gene expression (Lu *et al.* 2022). These metabolic pathways are involved specifically during terminal erythroid differentiation which corroborates with the stage of the altered phenotype seen in the Jak2 V617F dataset. The p53 pathway operates comparably, controlling the switch between proliferation and differentiation, in addition to coordinating GATA1 expression in erythrocytes (Le Goff *et al.* 2021). In fact, aberrant activation of the p53 pathway has been associated with anaemia in ribosomopathies (Le Goff *et al.* 2021). Conversely, in Jak2 V617F, an inverse scenario might be in effect, potentially driving increased terminal differentiation. Finally, the regulation of cell cycle speed and critically, the rate of transition to S phase has recently emerged as another regulatory mechanism governing erythroid differentiation and output (Socolovsky 2022).

It is important to stress that diffGEK also recovered differences in genes that were ‘missed’ by differential expression. Some of these genes, such as Cdkn1a, Bbc3 and Dusp1 belong to the p53 pathway that was enriched amongst differentially expressed genes; and likewise alterations in the kinetics of Nfkb1a, Cdkn1a were identified that belong to the cell cycle G1/S transition pathway. In addition other genes belonged to pathways that were not picked up by gene ontology on differential expression and warrant further exploration such as the intracellular transduction machinery downstream of the TNFR2 pathway and the CD40L pathway, which are targets commonly implicated in lymphocyte differentiation (extra pathways not shown). Globally, genes belonging to the same pathway demonstrated concordant kinetic changes, evidenced by strong enrichment for terms when looking at all genes with changes in either transcription, splicing or degradation. For example, genes with altered transcription rates were overrepresented for cholesterol and steroid metabolism, as well as the G1/S transition. Conversely, genes with altered splicing rates were enriched in immune signalling related pathways (CD40L, TNFR2, NFKB, and IL-1 signalling) and apoptotic terms. These consistent patterns amongst pathway members may imply that different pathways are subjected to alterations in different processes, i.e. transcription, splicing, degradation.

More broadly, this analysis underscores the value of exploring gene expression dynamics through scRNA-seq as an adjunct layer of comparison, to capture nuances that might be missed when solely examining differences in spliced mRNA abundance, highlighting new genes belonging to existing candidate pathways or identifying new candidate pathways altogether.

### 3.3 Transcriptional kinetics in Ezh2 KO myelopoiesis

**Figure 3.**
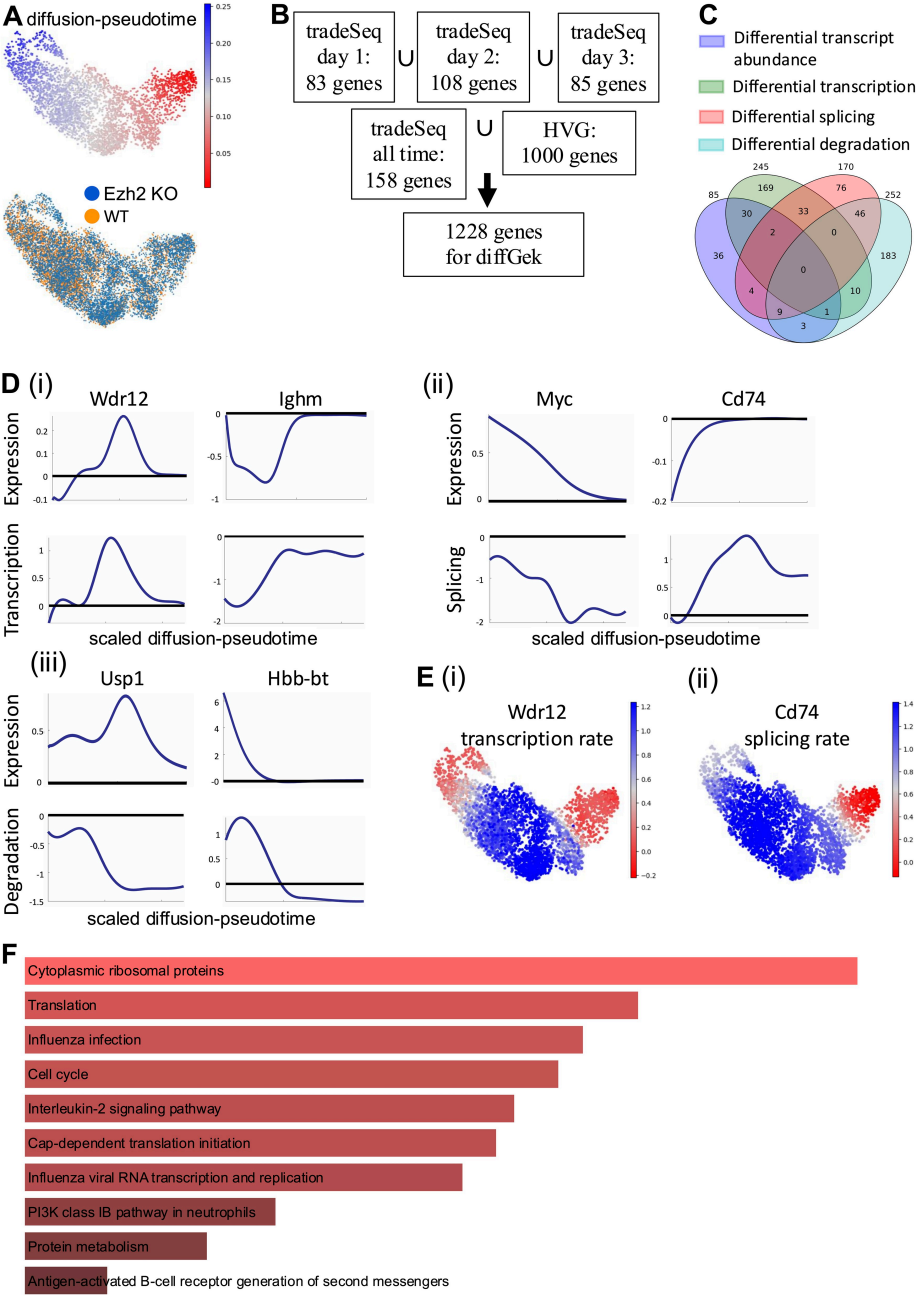
Application of the diffGEK pipeline to myelopoiesis in Ezh2 KO vs WT mice (A) UMAP showing diffusion pseudotime and conditions as adapted from the original manuscript (Liu *et al*. 2021). (B) Choice of genes for the diffGEK pipeline. The diffGEK pipeline needs the HVGs, but we also included DE genes as a way to double-check the tool performance. (C) Overlap between the differentially expressed genes and the diffGEK significant genes, sorted by rate type. (D) (i-iii) Examples of genes with differential kinetic rates (transcription, splicing or degradation respectively). For each gene, the panels represent the log2 fold change between the mutant and the wild type expression (top) and the rates (bottom). (E) Example of the log2 fold change of the transcription rate of Wdr12 (i) and the splicinng rate of Cd74 (ii). (F) Gene ontology of the diffGEK significant genes (that is, all but those classified as M1). Source: EnrichR, BioPlanet gene set. Adjusted *P-*values < 0.0001

Next, we applied our framework to another published scRNA-seq dataset which followed the combinatorial effects of two oncogenic mutations, $\mathrm{NRasG}12D^{+/-}$ and $\mathrm{Ezh}2^{ko/ko}$ on driving perturbed haematopoiesis across three timepoints (T1, T2, and T3) (Liu *et al.* 2021). By T2, the mice had developed myeloproliferative neoplasm, which by T3 had progressed to acute myeloid leukaemia, characterized by arrested myeloid differentiation and the accumulation of immature myeloid cells. Based on this, we narrowed down our analysis to cells belonging to the myeloid trajectory (Fig. 3A) and applied the diffGEK pipeline to interrogate differences between the wild-type and mutant state. Overall, the myeloid trajectory input into diffGEK consisted of 11 984 cells, derived from two sets of paired mutant and wild type mice. Of note, this dataset was deeply sequenced, with a median of 3144 transcripts per cell, enabling reliable capture of unspliced reads. Given the presence of a time course, we selected genes expressed differentially on the trajectory via tradeSeq, comparing conditions one time point at a time, and for all timepoints together. Altogether, we ran diffGEK for 1228 genes, including the top 1000 HVGs (Fig. 3B). Since the most pronounced alterations to myeloid differentiation manifested at T3, we explored the results of diffGEK on the wild-type and mutant trajectory at this timepoint.

diffGEK recovered 667 genes with differential kinetics of which 428 genes had alterations in one parameter only, and 239 had changes in more than one parameter (Fig. 3C). This highlights two key distinctions from the analysis of the pre-leukaemic Jak2 dataset: (i) there were globally a higher number of genes with differential kinetics, and (ii) there were genes with multiple parameter changes—which may be aligned with increased dysregulation in the leukaemic state. Moreover, hypergeometric test again showed statistically significant overlap between genes identified to have distinct TSDr and altered transcript abundance (differentially expressed genes). For such genes that had altered TSDr and differential expression, diffGEK provides an interpretability layer to understand the kinetic process driving the altered transcript abundance, as seen by the examples in Fig. 3D. As in the previous case, kinetic differences can be visualized along the differentiation trajectory, to pinpoint the cell types where they are more pronounced, which are gene-dependent (Fig. 3E).

Next, we explored the gene ontology of those genes identified with differential TSDr (Fig. 3F). Pathway enrichment analysis of genes identified by diffGEK demonstrated significant enrichment for a number of relevant terms. Pathways with the highest enrichment score, included: translation and ribosomal proteins, whose intricate regulation is perturbed in myeloid leukaemias and is a driver for protein metabolism that promotes elevated cell cycling which was another highly enriched term. In addition, other pathways with high enrichment consisted of interleukin-2 signalling, which has been reported to be a key signalling pathway required for the survival of leukaemia initiating cells in chronic myeloid leukaemia (Kobayashi *et al.* 2014); moreover, this has been shown to be an early change. The Class IB PI3K pathways, and Aurora B pathways were also enriched and have published roles in driving AML (Darici *et al.* 2020), and AML t(8; 21) (Qi *et al.* 2019) respectively.

Taken together, this analysis highlights that diffGEK is able to uncover changes in TSDr, early in transformation before they are picked up by differential expression, in important genes belonging to candidate pathways associated with myeloid leukaemia.

## 4 Discussion

Here we present, diffGEK, a comparative technique which leverages both spliced and unspliced mRNA counts, to extract trajectory-based differential gene transcription, splicing and degradation rates between conditions. diffGEK builds on the current suite of scRNA-seq comparative tools by enabling researchers to factor in the continuous nature gene expression profiles inherent to differentiation trajectories, while diving deeper to deliver interpretability into the gene expression kinetics changes that drive altered transcriptional profiles. We propose for diffGEK to be applied to the end of bioinformatic pipelines as an adjunct to DGE, where it can provide a more granular approach to extract differences between conditions which may not precipitate into altered mRNA abundance, while providing additional interpretability.diffGEK derives TSDr from conventional scRNA-seq datasets, without the need for metabolic labelling experiments, or alterations to single cell sequencing protocols. This is achieved through leveraging unspliced reads, which are a largely underused modality in scRNA-seq. One of the major attractions of utilizing the unspliced reads layer is that the added information provided is a low-hanging fruit which is essentially available at no additional cost as it is extracted from the raw scRNA-seq counts. It is worth noting that existing scRNA-seq technologies are not optimized for capturing intronic reads with high efficiency. This is due to their heavy reliance on priming methods, which sequence either the 3′ or 5′ ends of genes while neglecting the intervening regions. However, the detection of intronic reads can be plausibly attributed to secondary priming positions of oligo dT onto short stretches of poly-A sequences within introns. Despite not being optimized for the capture of intronic reads, scRNA-seq protocols are capable of recovering this modality in significant depth. In fact, an analysis of single-cell RNA-seq datasets derived from a range of leading protocols such as SMART-seq2, STRT/C1, inDrop and 10× Genomics Chromium found that 15%–25% of all captured reads were unspliced sequences. We must acknowledge a caveat concerning scRNA-seq alignment frameworks, which primarily attribute the presence of introns as a consequence of incomplete processing of pre-mRNA. However, it is important to recognize that other mechanisms, such as intron retention, can also contribute to the presence of intronic sequences in fully processed cytoplasmic mRNA molecules. Consequently, these intronic sequences may be erroneously assigned as unspliced reads. However, to address this concern, La Manno *et al.* conducted metabolic labelling to show that the number of unspliced reads recovered by scRNA-seq correlate with metabolically labelled nascent mRNA molecules, and the level of scRNA-seq spliced counts, suggesting that these intron-containing molecules recovered by scRNA-seq predominantly represent unspliced precursor mRNAs; this emphasizes the considerable potential for the development of methodologies explicitly designed to enhance the retrieval of unspliced and intronic sequences. Moreover, the integration of approaches such as single-cell metabolic labelling, which provide ground-truth measurements of unspliced mRNA, could, similarly to Volteras *et al.* (2024), further enrich the pipeline and amplify the signal-to-noise ratio. It is important to note that our diffGEK analysis fully supports the notion that unspliced reads extracted even with current sequencing technologies and alignment methods, are sufficient to provide meaningful gene expression kinetic rates. These TSDr effectively explain differentially expressed genes and enrich for relevant biological pathways. This solidifies the inherent value of unspliced counts as genuine biological signal, thereby extending the usability of this analysis to the majority of scRNA-seq datasets.

At its core, diffGEK builds upon and refines the RNA velocity framework. The significant innovation introduced by RNA velocity, in modelling differentiation dynamics through the relative abundance of spliced and unspliced reads, has come with a number of technical issues. Ever since the seminal 2019 article by La Manno *et al.* several subsequent studies have tackled its limitations. These advancements include accommodating varying gene activation states along a trajectory (as demonstrated in Bergen et al.), employing latent space representations to counteract inference errors due to count noise, especially within unspliced counts (Qiao and Huang, 2021), integrating cell-specific kinetic rates to accommodate kinetic enhancements (Barile *et al.* 2021), and addressing potential alterations in transcriptional kinetics within differentiating systems (as indicated in a recent publication). In this work, we particularly focused on comparing genes with kinetic changes (differential TSDr) among conditions. To this aim, we expanded upon the existing RNA velocity paradigm by enabling cell-specific kinetic rates within a predetermined trajectory based on precomputed pseudotemporal cell order. Contrary to the approach in cellDancer (Li *et al.* 2024), which assumes independent kinetic rates for each cell, our approach smoothed the rates over a trajectory. Despite being a strong assumption, the method proved to be significantly more stable, especially considering that in our test datasets, independent cell kinetics failed to recapitulate the known biology (data not shown).

diffGEK provides a flexible framework that can be used downstream of any dimensionality reduction and trajectory inference method. As it is agnostic to the dimensionality reduction and trajectory inference methodology, the approach scales from simple to complex trajectories with multiple bifurcations: diffGEK only requires the original spliced and unspliced expression count matrix of the individual cells, estimated pseudotimes, and a hard assignment of the cells to the lineages. The flexibility provided by diffGEK to work downstream of any trajectory inference technique is crucial, as trajectory-based DE is often the final (or near final) step in a much longer analysis pipeline. Nevertheless, it is worth mentioning that our method, while allowing for flexibility, as with other terminal scRNA-seq analytical tools still remains strongly dependant on the pre-defined trajectories and pseudotime. A different definition of trajectories and pseudotime could identify different kinetically variable genes. Furthermore, we relied on the assumption that the TSDr can be modelled with smoothed splines, differently from our previous attempt to model them with completely discontinuous transitions (Barile *et al.* 2021). The discontinuous transitions can still be obtained in our current framework upon increasing the number of spline nodes, but has a computational cost and an overfitting risk. Another limitation is that diffGEK cannot rank genes, only classify them, thus failing to provide a hierarchy of genes for which there is a greater confidence of having differential kinetics. Furthermore, the pipeline’s results are sensitive to the number of genes that are picked for the analysis: we experienced problems when this number increases by 10 fold (10 000 genes instead of 1000), because an intra-pipeline smoothing step is affected by the presence of noisy genes; on the other side, a small number of genes may overlook important differences present in the excluded genes. Nevertheless, our pipeline has proven to bring insights in experiments with multiple conditions and allows for further improvement, such as unsupervised learning of trajectories and latent time, and higher computational efficiency.

Our tool has been optimized and coded for comparison of two conditions, but it can easily modified for multiple conditions, provided that the optimization parameters are re-optimized similarly to what described here. Note, though, that the computational power scales with the number of compared conditions in a power-like fashion.diffGEK offers a range of benefits for biological interpretation. It offers an explanatory lens into the root causes of differential transcript abundance observed in DGE, providing a finer resolution to guide potential corrective strategies. In addition to enhancing interpretability, diffGEK probes beyond surface level spliced mRNA expression to uncover nuanced differences that may not be reflected in differential transcript abundance. In doing so this method can facilitate the identification of potential new gene candidates within enriched pathways or the discovery of entirely novel candidate pathways that could be instrumental in driving the observed altered phenotype. It is important to stress that the interpretability afforded by diffGEK operates on two levels. It not only clarifies the nature of kinetic alterations (transcription, splicing, and degradation) but also identifies specific pseudotime locations along the trajectory where substantial differences arise. This understanding has the potential to facilitate targeted therapeutic approaches directed at specific cell types and distinct processes of the mRNA lifecycle. Indeed a range of RNA-based and protein-based modulators have emerged which employ an array of strategies to modulate transcription, splicing and degradation rates. However, the majority of these modifiers lack gene-specific action, exerting their effects globally across multiple genes. We anticipate that diffGEK may provide valuable insights into the pathophysiology of various solid tumours and haematological malignancies carrying mutations in genes that modulate TSDr. Notably, mutations in spliceosomal genes such as SF3B1, U2AF1, SRSF2, ZRSR2, and the RNA-stabilizing methyltransferase METTL3 are specifically enriched in haematological disease. This is particularly pertinent in the haematological system as conventional analysis suites for DGE or alternative splicing analysis have provided limited understanding of disease mechanisms. Nevertheless, our analysis of the two datasets presented in this study reveals that mutations in genes unrelated to transcription, splicing, and degradation can also influence TSDr. This highlights the broader applicability of diffGEK in exploring various cell-intrinsic mutational contexts, as well as cell-external factors like pharmacological drugs and cell-reprogramming cytokines, which drive diverse cellular states.

## Supplementary Material

btaf316_Supplementary_Data

## Data Availability

For this study we did not produce new data. Jak2 data was taken from Isobe *et al.* 2023. Ezh2 data was taken from Liu *et al.* (2021). Simulated data are available on request. All the codes and an explicatory vignette have been uploaded to GitHub: https://github.com/mebarile/transcriptional_kinetics.

## References

[btaf316-B1] Barile M , Imaz-Rosshandler I, Inzani I et al Coordinated changes in gene expression kinetics underlie both mouse and human erythroid maturation. Genome Biol 2021;22:197–40. 34225769 10.1186/s13059-021-02414-y PMC8258993

[btaf316-B2] Bergen V , Lange M, Peidli S et al Generalizing RNA velocity to transient cell states through dynamical modeling. Nat Biotechnol 2020;38:1408–14. 32747759 10.1038/s41587-020-0591-3

[btaf316-B3] Darici S , Alkhaldi H, Horne G et al Targeting PI3K/Akt/mTOR in AML: rationale and clinical evidence. J Clin Med 2020;9. 10.3390/jcm9092934 PMC7563273 32932888

[btaf316-B4] Fischer DS , Fiedler AK, Kernfeld EM et al Inferring population dynamics from single-cell RNA-sequencing time series data. Nat Biotechnol 2019;37:461–8. 30936567 10.1038/s41587-019-0088-0 PMC7397487

[btaf316-B5] Gao M , Qiao C, Huang Y. UniTVelo: temporally unified RNA velocity reinforces single-cell trajectory inference. Nat Commun 2022;13:6586. 36329018 10.1038/s41467-022-34188-7 PMC9633790

[btaf316-B6] Isobe T , Kucinski I, Barile M et al Preleukemic single-cell landscapes reveal mutation-specific mechanisms and gene programs predictive of aml patient outcomes. Cell Genom 2023;3:100426. page 38116120 10.1016/j.xgen.2023.100426 PMC10726426

[btaf316-B7] Kobayashi CI , Takubo K, Kobayashi H et al The il-2/cd25 axis maintains distinct subsets of chronic myeloid leukemia-initiating cells. Blood 2014;123:2540–9. 24574458 10.1182/blood-2013-07-517847

[btaf316-B8] La Manno G , Soldatov R, Zeisel A et al RNA velocity of single cells. Nature 2018;560:494–8. 30089906 10.1038/s41586-018-0414-6 PMC6130801

[btaf316-B9] Lange M , Bergen V, Klein M et al CellRank for directed single-cell fate mapping. Nat Methods 2022;19:159–70. 35027767 10.1038/s41592-021-01346-6 PMC8828480

[btaf316-B10] Le Goff S , Boussaid I, Floquet C et al p53 activation during ribosome biogenesis regulates normal erythroid differentiation. Blood 2021;137:89–102. 32818241 10.1182/blood.2019003439

[btaf316-B11] Li S , Zhang P, Chen W et al A relay velocity model infers cell-dependent RNA velocity. Nat Biotechnol 2024;42:99–108. 10.1038/s41587-023-01728-5 37012448 PMC10545816

[btaf316-B12] Liu Y , Gu Z, Cao H et al Convergence of oncogenic cooperation at single-cell and single-gene levels drives leukemic transformation. Nat Commun 2021;12:6323. 34732703 10.1038/s41467-021-26582-4 PMC8566485

[btaf316-B13] Lönnberg T , Svensson V, James KR et al Single-cell RNA-seq and computational analysis using temporal mixture modelling resolves Th1/tfh fate bifurcation in malaria. Sci Immunol 2017;2. 10.1126/sciimmunol.aal2192 PMC5365145 28345074

[btaf316-B14] Love MI , Huber W, Anders S. Moderated estimation of fold change and dispersion for rna-seq data with deseq2. Genome Biol 2014;15:550. 25516281 10.1186/s13059-014-0550-8 PMC4302049

[btaf316-B15] Lu Z , Huang L, Li Y et al Fine-tuning of cholesterol homeostasis controls erythroid differentiation. Adv Sci 2022;9:e2102669. 10.1002/advs.202102669 PMC8805577 34739188

[btaf316-B16] Qi J , Gao X, Zhong X et al Selective inhibition of aurora a and b kinases effectively induces cell cycle arrest in t(8; 21) acute myeloid leukemia. Biomed Pharmacother 2019;117:109113. 31207577 10.1016/j.biopha.2019.109113

[btaf316-B17] Qiao C , Huang Y. Representation learning of RNA velocity reveals robust cell transitions. Proc Natl Acad Sci USA 2021;118:e2105859118. 34873054 10.1073/pnas.2105859118 PMC8670433

[btaf316-B18] Qiu X , Mao Q, Tang Y et al Reversed graph embedding resolves complex single-cell trajectories. Nat Methods 2017;14:979–82. 28825705 10.1038/nmeth.4402 PMC5764547

[btaf316-B19] Qiu X , Zhang Y, Martin-Rufino JD et al Mapping transcriptomic vector fields of single cells. Cell 2022;185:690–711.e45. 35108499 10.1016/j.cell.2021.12.045 PMC9332140

[btaf316-B20] Robinson MD , McCarthy DJ, Smyth GK. edgeR: a bioconductor package for differential expression analysis of digital gene expression data. Bioinformatics 2010;26:139–40. 19910308 10.1093/bioinformatics/btp616 PMC2796818

[btaf316-B21] Socolovsky M. The role of specialized cell cycles during erythroid lineage development: insights from single-cell rna sequencing. Int J Hematol 2022;116:163–73. 35759181 10.1007/s12185-022-03406-9 PMC12302956

[btaf316-B22] Tiberi S , Meili J, Cai P et al DifferentialRegulation: a bayesian hierarchical approach to identify differentially regulated genes. Biostatistics 2024;25:1079–93. 38887902 10.1093/biostatistics/kxae017 PMC11639160

[btaf316-B23] Van den Berge K , Roux de Bézieux H, Street K et al Trajectory-based differential expression analysis for single-cell sequencing data. Nat Commun 2020;11:1201. 32139671 10.1038/s41467-020-14766-3 PMC7058077

[btaf316-B24] Volteras D , Shahrezaei V, Thomas P. Global transcription regulation revealed from dynamical correlations in time-resolved single-cell RNA sequencing. Cell Syst 2024;15:694–708.e12. 39121860 10.1016/j.cels.2024.07.002

