# Supplementary material for: diffGEK: differential gene expression kinetics
Source: Bioinformatics. 2025 Jun 10;41(6):btaf316. doi: 10.1093/bioinformatics/btaf316 (PMC12198498; doi:10.1093/bioinformatics/btaf316)
Supplement: btaf316_Supplementary_Data [file btaf316_supplementary_data.pdf]

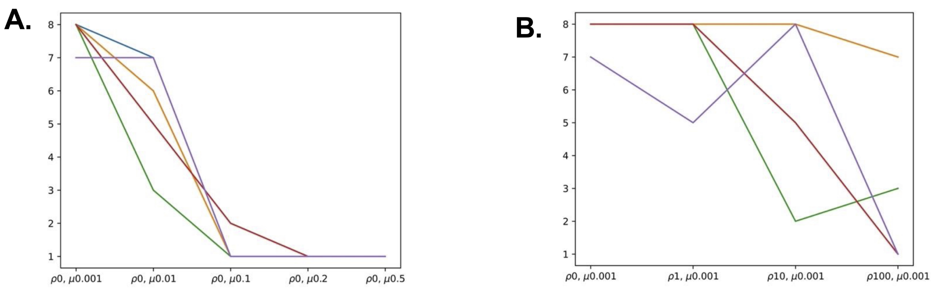

Supplemental Figure 1. Calibration of the regularization parameters (A) Calibration when ρ is fixed and μ varies. The y axis shows the chosen model (M1-M8 in the main text); each line corresponds to a gene. We see in general that for a very small error, the models with higher number of significantly different kinetics are picked. For very big errors, the models with few or no significantly different kinetics are picked. (B) Calibration when μ is fixed and ρ varies. The y axis shows the chosen model (M1-M8 in the main text); each line corresponds to a gene. Smaller regularization terms for the splines correspond to overfitted models, that can reproduce even small differences among conditions, and the other way around. However, it is also clear that the two parameters have a combined effect and should be calibrated together.
